# Supplementary material for: eeeHive: a new HF RFID-based automated behavioral monitoring system enabling high spatiotemporal resolution tracking of group-housed animals
Source: Front Behav Neurosci. 2026 Oct 7;20:1861925. doi: 10.3389/fnbeh.2026.1861925 (PMC13683769; doi:10.3389/fnbeh.2026.1861925)
Supplement: Supplementary file 1 [file Data_Sheet_1.pdf]

Supplementary Material

Supplementary Animation 1. Tracking of group-housed mice using eeeHive 2D.

Time-lapse visualization of positional detections for all mice over the 7-day experimental period, based on antenna-derived spatial data. Dark background indicates dark period: lights on at 8:00, lights off at 20:00.

Supplementary Animation 2. Tracking of group-housed marmosets using eeeHive FLEX.

Time-lapse visualization of antenna-access events in common marmosets. Recordings from Week 1, Week 2, and Week 5 are shown to illustrate temporal changes in access patterns. Dark background indicates dark period: lights on at 7:00, lights off at 19:00. NEST, FOOD, etc. indicate antenna locations (multiple nest boxes, feeders, water stations). The videos are trimmed during the dark period because they rarely move at night.

Supplementary Material

Supplementary Figure 1.

A

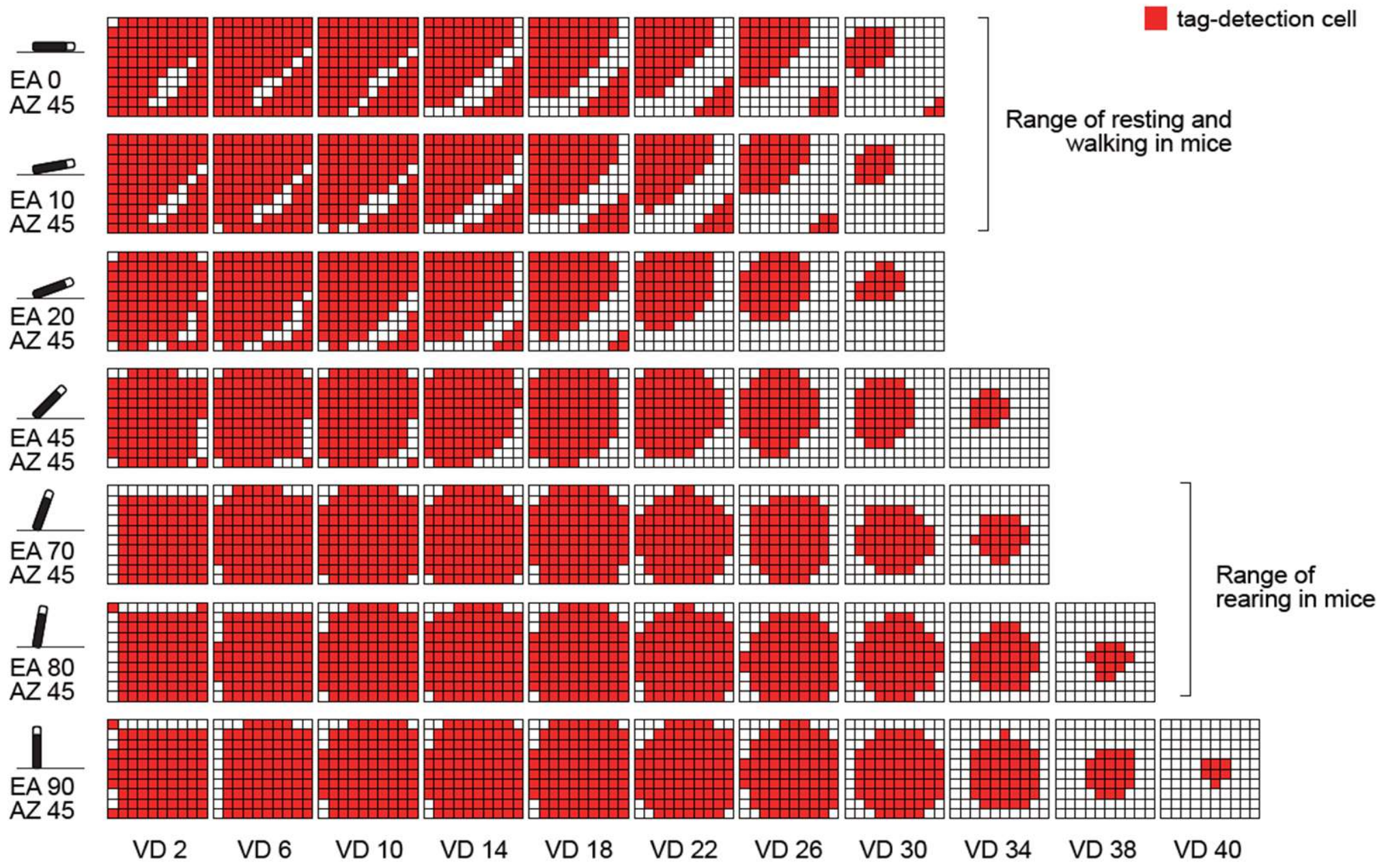

B

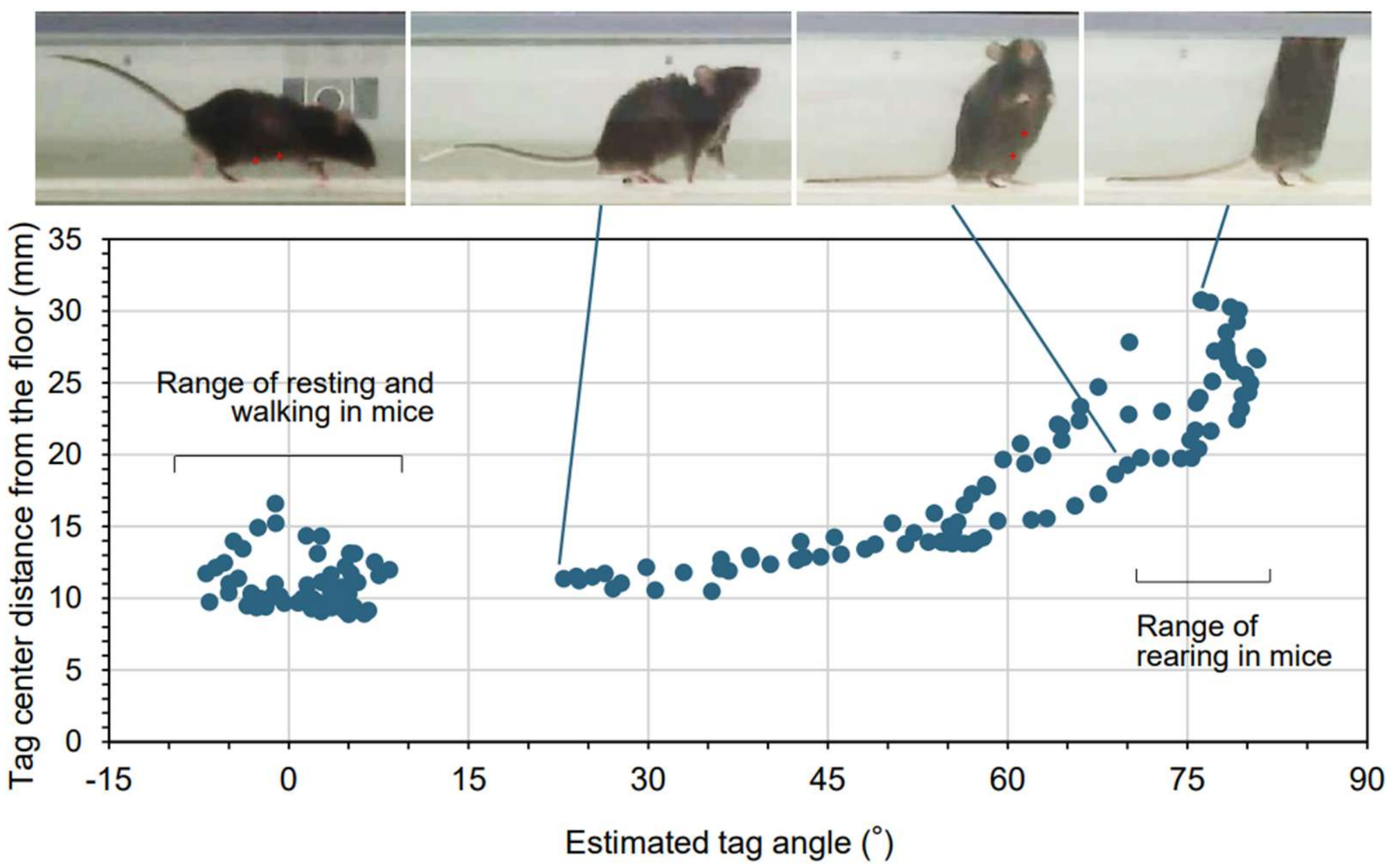

Supplementary Figures for the Tag Detection Range.

(A) Tag detection performance across spatial configurations. Detection results are shown for all elevation angles (EA 0–90°) and vertical distances (VD 2–40 mm) under an azimuth angle of 45°.

(B) Approximate position of the implanted tag in freely moving mice within the cage. Tag orientation and distance from the cage floor were estimated from video recordings of mice in different postures (walking, crouching, and standing) using MotoRater (TSE Systems, Germany). The distance from the floor (mm) was measured relative to the center of the estimated tag position. Tag angles for each posture were determined based on the contour of the ventral body surface.

Supplementary Material

Supplementary Figure 2.

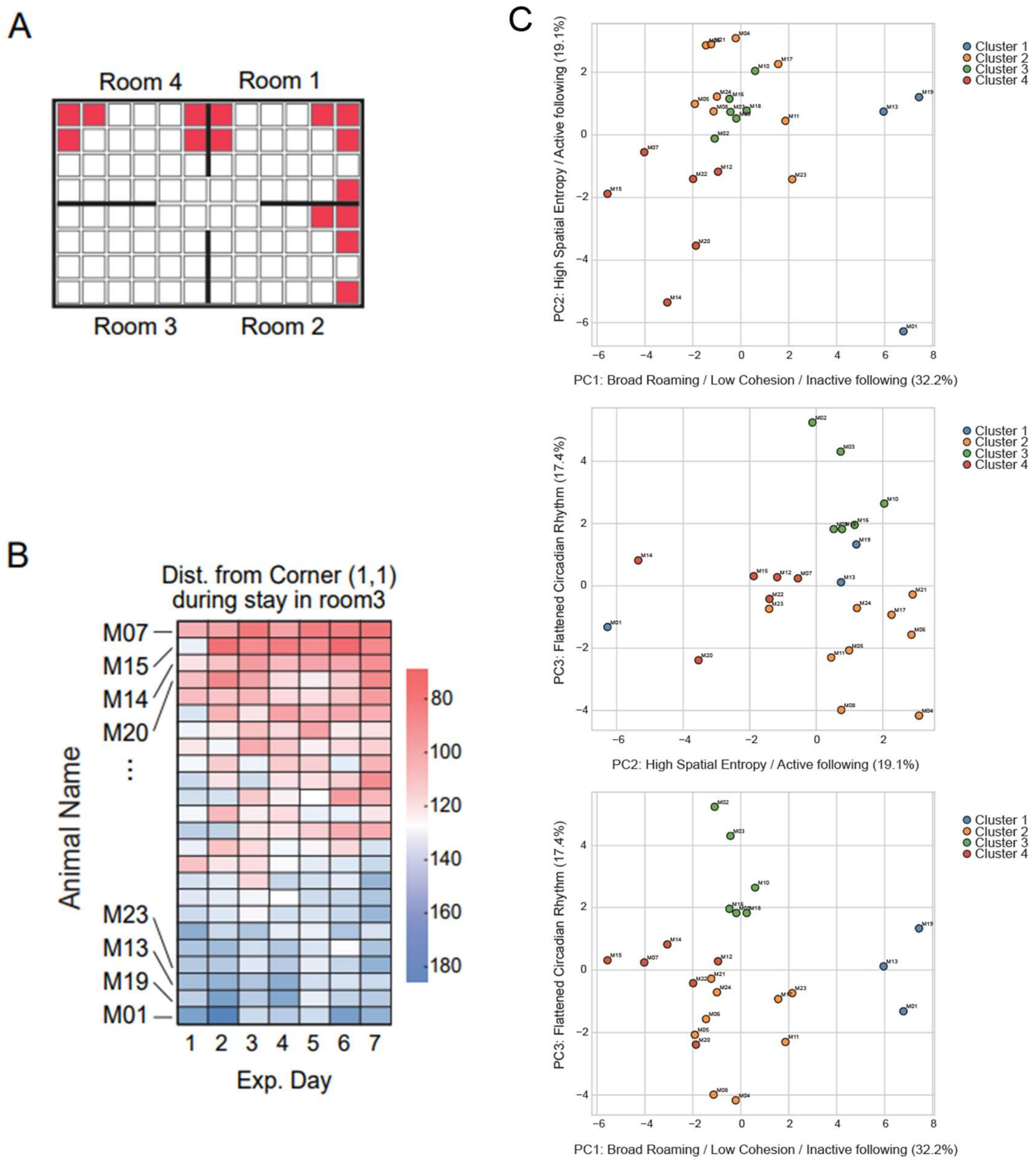

Supplementary Figures for the Mouse Experiment. (A) Distribution of urine observed on the final day of the experiment (Day 7). The schematic highlights approximate urine locations in red. (B) Day-by-day changes in Distance from Corner (DFC) for each individual across the experimental period. (C) The pairwise biplots of the three principal components.

Supplementary Figure 3.

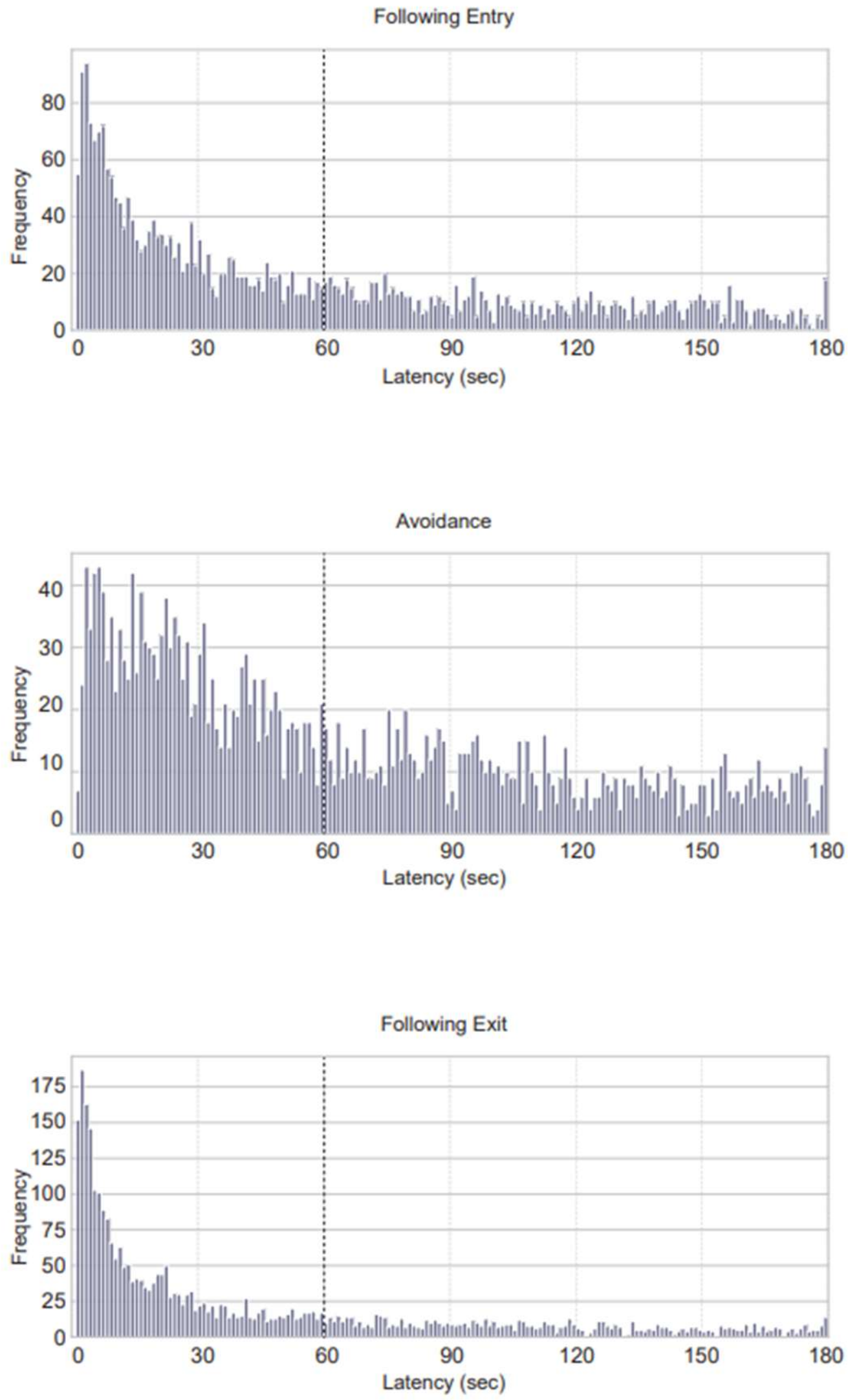

Supplementary Figures for the Marmoset Experiment. Top panel: Histogram of latencies for Following Entry (the time elapsed from when individual A enters Nest X until individual B enters the same Nest X), generated from all pair data. Middle panel: Histogram of latencies for Avoidance (the time elapsed from when individual B enters Nest X, where individual A is already present, until individual A exits), generated from all pair data. Bottom panel: Histogram of latencies for Following Exit (the time elapsed from when individual A exits Nest X until individual B exits the same Nest X), generated from all pair data.

Supplementary Figure 4.

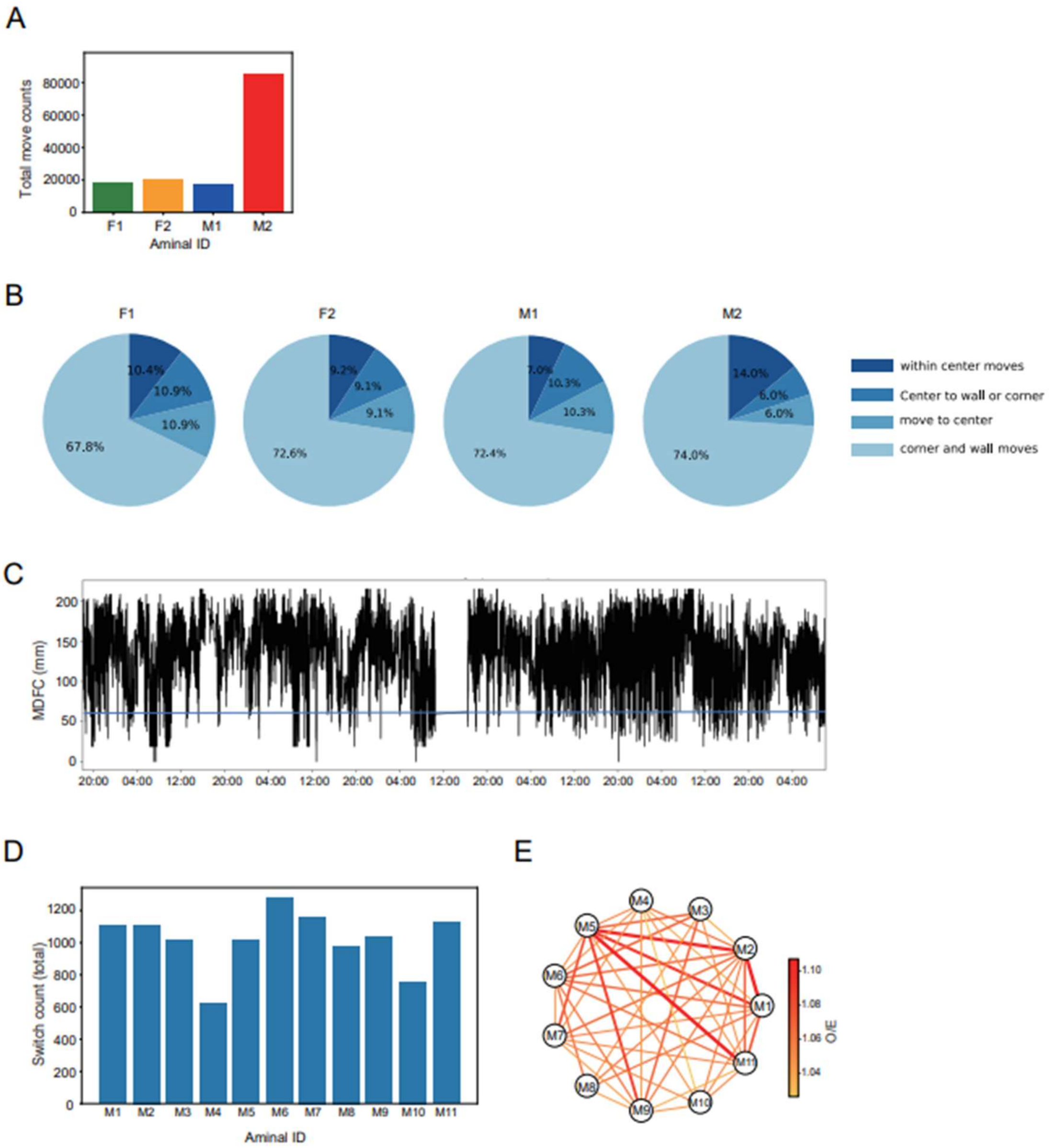

Supplementary Figures for the Aquatic Environment Experiment. (A) Total movement counts for each individual newt across the entire experimental period. (B) Movement categories. (C) Time series of group cohesion at 1-Hz resolution (1-s intervals), quantified as the mean distance from centroid (MDFC). The y-axis indicates MDFC (mm). They were fed around 18:00. (D) Total trafficking (compartment switching) counts per fish over the recording period. (E) Pairwise co-presence network. Undirected network constructed from pairwise same-compartment O/E ratios. Edges represent significant pairwise co-presence relationships (permutation test, $p < 0.05$).

Supplementary Information 1. Supplementary Methods for Evaluation of Tag Read Range and Processing Speeds.

Because the coil of the cylindrical tag is wound around a ferrite core with high magnetic permeability, the magnetic flux is guided and concentrated within the coil even when its direction does not perfectly align with the longitudinal axis of the tag, enabling stable tag detection. Due to this effect, accurate estimation of the detection range by simple simulation is difficult; therefore, all evaluations in this study were performed experimentally.

For tag placement, seven elevation angles (0°, 10°, 20°, 45°, 70°, 80°, and 90°) and two azimuth angles (0° and 45°) were tested. The vertical distance from the antenna surface was set to 11 levels ranging from 2 to 40 mm (2, 6, 10, 14, 18, 22, 26, 30, 34, 38, and 40 mm). The horizontal position on the antenna surface was defined by dividing a 50 × 50 mm area into a 10 × 10 grid (100 cells). For all combinations of these conditions, whether the tag ID could be successfully read was recorded.

For the measurements, a 2-mm-thick transparent acrylic plate with a 5-mm grid printed on a transparent film was placed on the tile-type antenna to allow visualization of grid positions. A tag with a fixed elevation angle (using resin) was placed on the grid and moved horizontally while maintaining a constant azimuth angle. Tag readability was evaluated when the bottom end of the tag was positioned at the center of each grid cell, and the percentage of successful reads to total cells was defined as the Detection Area Coverage (%). The vertical distance was adjusted by stacking the 2-mm-thick acrylic plates, and Detection Area Coverage (%) was recorded for each condition.

The same procedure was applied to the ring-type antenna, except that cells corresponding to regions outside the circular coil were excluded from the analysis. In addition, tag reading distances in air, freshwater (hardness: 60–80 mg/L), and a 3.5% NaCl solution (Wako, Japan) were compared using the ring-type antennas. A ring-type antenna was lowered toward a tag fixed vertically ~2 cm above the bottom of a 3-L beaker. For liquid conditions, the antenna's magnetic field was thought to be fully submerged. Three independent antennas were tested.

Next, the polling cycle time was measured for 24 tile-type antennas in the 2D module and 24 ring-type antennas in the FLEX module. A single tag was placed at the center of each antenna (elevation angle 90°, vertical distance 0 mm), and the number of tags was gradually increased while measuring the polling cycle time. To evaluate the effect of anti-collision processing, multiple tags (up to 13) were placed on a single antenna, and the polling cycle time was measured for each condition. From the measured polling cycle time, the polling time per antenna and the tag read time per tag were calculated.

Supplementary Information 2. Validation of eeeHive 2D positional recording using manual video-based scoring

To validate the accuracy of the eeeHive 2D module in determining mouse positions within a cage, four adult male C57BL/6J mice with tags implanted subcutaneously in the abdomen were housed together in a cage (300 mm W × 200 mm D × 150 mm H). Beginning on the second day of group housing, their behavior was simultaneously recorded for 24 h using both the eeeHive 2D module positioned beneath the cage and an overhead video camera.

Analysis frames were systematically extracted from the video at approximately 10-min intervals throughout the 24-h period, yielding a total of 143 frames. The positions of all four mice were manually determined in each frame. Individual mice were identified by shaving patterns: no shaving, shaving of the upper trunk, shaving of the lower trunk, and shaving of the dorsal region.

Because the drinking area was located outside the main cage area and accessed through a tunnel, observations in which a mouse was present in the drinking area were excluded. Consequently, a total of 547 comparisons were made between antenna positions determined by manual video scoring and the corresponding antenna positions recorded by the eeeHive 2D system.

To facilitate manual positional scoring, a grid corresponding to the antenna layout of the eeeHive 2D module was superimposed onto each video frame, and the antenna occupied by each mouse was determined (Figure S2).

The eeeHive 2D module outputs a single antenna position for each time point after chattering correction. In contrast, mice frequently occupied the boundaries between adjacent antennas, and the exact position of the tag implanted in the abdomen could not always be uniquely determined from the overhead video alone. Therefore, during manual scoring, the most likely antenna position, together with the second and third candidate antenna positions, was recorded for every observation.

All manual scoring procedures described above were performed using a dedicated web browser-based application developed in Python to ensure a standardized evaluation procedure.

Comparison of the manually scored positions with the corresponding antenna positions recorded in the eeeHive 2D logs at the same time points showed an agreement rate of 50.3% (275/547) when only the first candidate was considered. The agreement increased to 87.8% (480/547) when the second candidate was accepted and to 94.3% (516/547) when up to the third candidate was accepted.

Cases that agreed with the first candidate alone mainly corresponded to situations in which the entire body of the mouse was located within a single antenna region. In contrast, cases requiring multiple candidate positions were typically those in which the mouse body extended across two or more adjacent antenna regions. Considering that a mouse's body often spans two or more antennas, the agreement rate described above was considered sufficiently high.

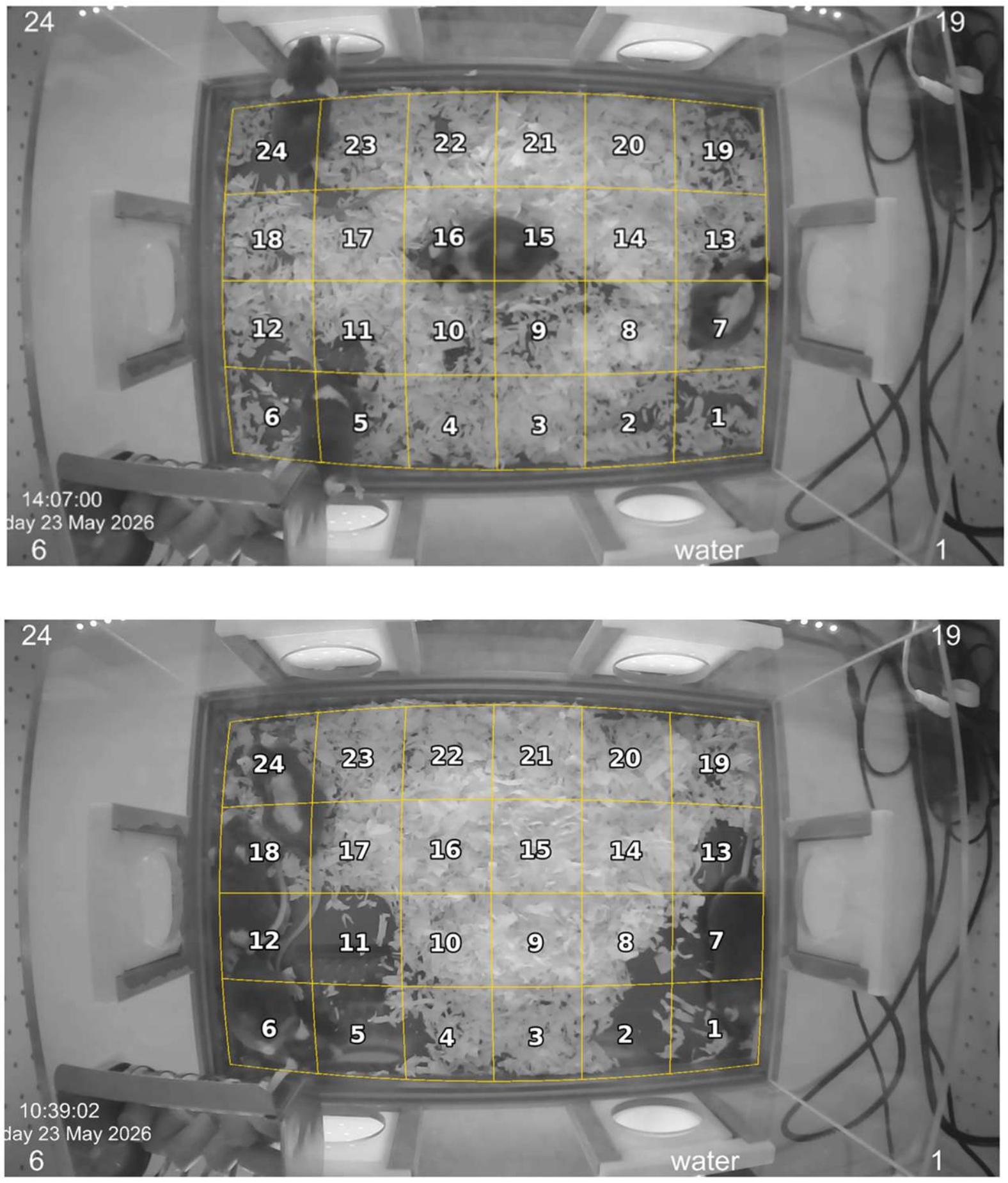

Figure S2. Representative screenshot of the validation interface used for manual scoring. A grid corresponding to the antenna layout of the eeeHive 2D module was superimposed on synchronized overhead video images. For each mouse, the most likely antenna position (first candidate), together with the second and third candidate positions when necessary, was recorded.

Supplementary Information 3. Tag read success rate per polling cycle using the validation dataset

Tag read success rate per polling cycle was evaluated using the 24-h validation dataset described in Supplementary Information 2.

To compare tracking performance under different activity levels, four representative time windows were selected: early light phase (08:32:55-08:39:26), mid light phase (14:59:53-15:08:53), early dark phase (20:58:26-21:05:27), and mid dark phase (02:38:47-02:44:59). Time windows were selected such that none of the four mice entered the external water compartment for at least six consecutive minutes, as confirmed by video observation.

For each time window, 1,000 consecutive polling cycles were analyzed. For each tag, the number of polling cycles in which the tag was successfully detected was counted, and the tag read success rate per polling cycle was calculated.

The tag read success rates per polling cycle for each time window are summarized in Supplementary Table S3.

Across the four mice, the mean tag read success rates were 96.0% during the early light phase, 99.4% during the mid light phase, 78.9% during the early dark phase, and 78.4% during the mid dark phase. During the dark phase, individual tag read success rates ranged from 48.9% to 99.9%, presumably reflecting differences in locomotor activity and tag orientation.

Given that several polling cycles occur each second, these results indicate that the eeeHive 2D system maintained robust tag detection under both low- and high-activity conditions.

Table S3

Row Labels	M1	M2	M3	M4
Early light phase (8:30AM)	93.6%	99.1%	91.3%	100.0%
Mid light phase (3PM)	100.0%	100.0%	100.0%	97.4%
Early dark phase (9PM)	83.0%	99.6%	48.9%	84.0%
Mid dark phase (2:30AM)	99.9%	91.3%	55.8%	66.5%

Supplementary Information 4. Data completeness during the 7-day continuous recording of 24 mice

In the 7-day mouse experiment, 24 mice were simultaneously tracked using four eeeHive 2D modules. Because the polling interval of each board varied according to the number of tags present on that board at any given time, the polling-cycle-based read success rate used in the validation experiment (Supplementary Information 3) could not be directly applied to this dataset. Therefore, data completeness was evaluated as the proportion of one-second intervals in which each individual was detected at least once.

The analysis was performed using the raw RFID detection logs prior to chattering correction and missing-data imputation. Each detection record contained a millisecond-resolution timestamp, positional coordinates, and the individual ID, with each row representing a single RFID detection event. Timestamps were converted to one-second intervals, and the number of detections for each individual (M01-M24) was counted within each second. If an individual was detected one or more times within a given second, that second was regarded as successfully detected for that individual. Conversely, if no detection record was present for an individual during a given second, that second was defined as a missing second for that individual.

For each individual, the detection rate (%) was calculated as:

Detection rate (%) = (Number of detected seconds / Total analysis seconds) × 100

The mean detection rate per second across all 24 mice was 83.6%, with individual detection rates ranging from 74.9% to 90.0%. Detailed detection rates and numbers of missing seconds for each individual are summarized in Supplementary Table S4.

Supplementary Material

Table S4

Mouse ID	Total analysis seconds	Detected (sec)	Missing (sec)	Detected rate	Missing rate
M01	604800	544572	60228	90.04%	9.96%
M02	604800	515192	89608	85.18%	14.82%
M03	604800	516545	88255	85.41%	14.59%
M04	604800	453022	151778	74.90%	25.10%
M05	604800	506011	98789	83.67%	16.33%
M06	604800	511707	93093	84.61%	15.39%
M07	604800	508497	96303	84.08%	15.92%
M08	604800	459054	145746	75.90%	24.10%
M09	604800	485032	119768	80.20%	19.80%
M10	604800	513030	91770	84.83%	15.17%
M11	604800	474438	130362	78.45%	21.55%
M12	604800	503939	100861	83.32%	16.68%
M13	604800	506714	98086	83.78%	16.22%
M14	604800	525714	79086	86.92%	13.08%
M15	604800	495416	109384	81.91%	18.09%
M16	604800	502230	102570	83.04%	16.96%
M17	604800	516539	88261	85.41%	14.59%
M18	604800	524745	80055	86.76%	13.24%
M19	604800	530939	73861	87.79%	12.21%
M20	604800	542936	61864	89.77%	10.23%
M21	604800	470335	134465	77.77%	22.23%
M22	604800	502946	101854	83.16%	16.84%
M23	604800	515115	89685	85.17%	14.83%
M24	604800	510533	94267	84.41%	15.59%
Average				83.60%	16.40%

Supplementary Information 5. Supplementary information regarding the common marmoset diet

During weekdays, the marmosets were fed solid food (CMS-1M, CLEA Japan Co., Ltd., Tokyo, Japan) soaked in room-temperature water for 5 minutes. Twice a week, the diet was supplemented with either sweet potato and powdered milk (Pig Liquid Natural for piglets, Scientific Feed Laboratory Co., Ltd., Tokyo, Japan) or honey water (prepared by dissolving 2000 g of honey, 50 g of vitamin D, and 50 g of vitamin C in water to a total volume of 2000 mL).

Snacks such as boiled rice crackers (Osaka Maeda Seika Co., Ltd., Japan) and castella cakes (Bourbon Co., Ltd., Japan) were provided biweekly during handling for weight measurement. On weekends, the animals received soaked solid food on one day and unsoaked solid food on the other. As needed, the diet was further supplemented with Biofermin (Biosuri for animals, Toa Pharmaceutical Co., Ltd., Japan; 4 g), high-protein milk (Meiji Co., Ltd., Japan; 4 mL), high-protein milk powder (Tube Diet High-Protein, Morinaga Sanworld Co., Ltd., Japan; 1.7 g), or Recovery Gel (DietGel® Recovery, ClearH2O, USA; 56 g per cup).

Supplementary Material

Supplementary Information 6. Observational report by the marmoset caretakers

Period corresponding to behavioral data collection: April 18, 2023 – June 6, 2023

(A) Impressions of Behavior Toward Caretakers:

F1: Vigilant
F2: Friendly
M1: Cautious
M2: —
M3: Aggressive
M4: Vigilant

(B) Observational Impressions of Inter-individual Relationships During the Data Collection Period

F1: Good relationship with all other individuals (F1 dominant).

F2: Good relationship with F1 (F2 submissive), M1 (F2 submissive), M4 (F2 dominant). Poor relationship with M2 and M3.

M1: Good relationship with all other individuals (M1 dominant, except for F1, who is of equal status).

M2: Good relationship with F1 (M2 shows affiliative/dependent behavior), M1 (M2 submissive), M4 (M2 dominant). Poor relationship with M3 (M2 submissive), F2

M3: Good relationship with F1 (M3 affiliative), M1 (M3 rather subordinate at the beginning), M4 (M3 dominant). Poor relationship with M2 and F2 (M3 dominant)

M4: Good relationship with all other individuals (M4 submissive)

(C) Notes on Social Relationships and Animal Room Conditions (Including Pre- and Post-Experiment)

Until late January 2023, F1 accepted both M2 and M3 (embracement, mating).

January 18, 2023

M3 mated with F1; from this point until approximately May 2024, M3 and F1 formed a pair.

January 23, 2023

M2 attempted to mate with F1, but the attempt did not succeed because M3 interfered.

February 24, 2023

Food had been depleted and the group appeared hungry and irritated.
Feeding order observation: Observed order: M1 → F1 → M2 → M3 → F2.

March 23, 2023

M1 and F1 fought; hereafter, F1 came to act dominant.

April 3, 2023

Food ran out, F1 became irritated and threatened other individuals.
Feeding order observation: M1 ≈ F1 → (M2, M3, F2) → M4.
M1 was at the top, and F1 also competed strongly: F1 tried to eat first along with M1 and M1 scolded/reprimanded F1. However, F1 continued eating as if nothing had happened. M1 was strict toward M2, M3, and F2. M4 did not try to approach and stayed at the back.

April 14, 2023

M3 appeared to remain close to F1 and followed F1 constantly. F1 was presumed pregnant around this time (retrospective estimation).

April 17, 2023

M2 attempted mating with F1 but was noticed by M3 and interrupted, so the attempt ended unsuccessfully.

April 18, 2023

Room expanded. New individuals were also introduced (without chips).
**Initially, the housing area was divided into two compartments, with all tagged animals housed in one of the compartments. On the first day of data acquisition, the partition was removed to form a single contiguous space for behavioral tracking.*
M1 and F1 each threatened the newcomers, apparently to "teach"/establish hierarchy.
M2, M3, F2, and M4 did not show strong interest in the newcomers; they entered the new space and explored/played.
The newcomers (without tags) seemed afraid of individuals roughly up to F1, M1, M2, and M3. They showed submissive calls or hid in gaps/crevices. The newcomers did not appear to fear F2 or M4.

April 27, 2023

M3 followed F1 around. M2 continuously produced submissive vocalizations toward M3 and toward F1 (submissive, infant-like vocalizations were a typical vocal pattern observed as a sign of submission).

April 28, 2023

M2 repeatedly emitted submissive calls toward M3; M3 responded with threat vocalizations (video available). Previously, M2 and M3 often stayed together.

May 1, 2023

M2 produced submissive calls toward M3; M3 responded with threat vocalizations.

May 2–May 9, 2023 (Holidays)

Food and water were not replaced every day during the holiday period.

May 9, 2023

M2 produced submissive calls toward M3; M3 appeared angry/irritated in response.

May 17, 2023

The same pattern persisted: M2 continued submissive calling toward M3.

May 18, 2023

M2 produced submissive calls toward M3. M3 chased M2 while threatening; M2 fled.
This sequence (calling → chasing/threatening → fleeing) was repeated multiple times.

May 19, 2023

The same scene was observed again: M2 vocalizing toward M3, M3 threatening and chasing, M2 fleeing.

May 22, 2023

Box positions were changed (No. 5 & 6, and No. 15 & 16).

May 24, 2023

M3 repeatedly bit M2. M3 chased M2 persistently. Other individuals did not actively join or intervene, but appeared restless; the overall atmosphere was noisy/agitated.
M2 hid in narrow spaces (e.g., between the structure and wall) or behind logs.

June 7, 2023

M2 was bitten on the back by M3 and they were wrestling; the caretaker separated them.
After separation, M2 stayed quietly in the corner of the room, watching M3 cautiously.

June 8, 2023

As usual, M3 threatened; M2 remained vigilant and fled.

June 15, 2023

M2 provoked M3 using vocalizations; M3 chased M2. This did not escalate into a full wrestling fight, but the provoking/chasing sequence was observed frequently throughout the day.

June 23, 2023

A fight occurred between M2 and M3; M3 had a minor wound on the forehead. Even after being attacked, M2 quickly went back to approach M3 again.

June 26, 2023

M2 had a wound on the forehead (possibly due to fighting with M3).
M2 showed a decrease in body weight (366 g → 324 g). F1 showed a tendency toward weight gain.
Note: The repeated pattern was that M2 provoked, M3 became angry, and M2 fled; however, M2 did not appear to be trying to hide or escape permanently.

August 7, 2023

Food was almost empty; the individuals appeared very hungry (food had been replaced over the weekend).
F1 became angry if its feeding was disturbed and produced threat vocalizations; F1 was the dominant individual. Individuals other than F1 crowded around the three feeding sites and ate simultaneously. Unlike earlier periods, there was no longer an obvious pattern of allowing smaller individuals (like M4) to feed first. When M2 was eating, M3 came to interfere.

August 10, 2023

M2 appeared isolated and was vigilant toward M3. M2–M3 chasing continued. Previously, M2 had sometimes initiated interactions toward M3 while giving submissive calls, but recently the pattern was mainly initiated by M3. M3 approached the isolated M2, sat at a certain distance while asserting its presence, and then threat vocalizations and chasing began. M2 appeared highly wary of M3.

August 15, 2023 (Holiday period)

Food was empty; the group appeared hungry. M3 was seen chasing and threatening M2. F1 and M3 threatened others around the feeding area.
Because the group was restless/noisy overall, the caretaker entered and placed small amounts of dried food in multiple locations; the group ate and then calmed.

August 17, 2023

Food was empty; the group appeared hungry.
F1 initially monopolized feeding site A (Feeder 3,4) and ate first. Smaller individuals gathered at the site B(Feeder 1,2). M2 foraged for food that had dropped from the A/B feeding sites instead of feeding directly from the feeder boxes.

August 28, 2023

F1 threatened M1 (when feeding was interrupted).
While considering whether to install an additional wooden beam/log, all individuals showed interest at once except for M2.

September 5, 2023

F1 gave birth.

Supplementary Information 7. Quantitative estimation of LF RFID data loss caused by tag collisions

The analysis used the chattering-corrected positional dataset obtained from the 7-day mouse experiment. To estimate the information loss that would occur in an LF RFID system without anti-collision capability, a single-tag read constraint was applied independently to each time point and antenna.

When only one mouse occupied an antenna field, its positional record was considered retained. When two or more mice simultaneously occupied the same antenna field, only one positional record was considered retained, whereas the remaining records were counted as lost. The percentage loss at each occupancy level was calculated relative to the total number of records.

This analysis does not consider potential information loss attributable to differences in polling rate, antenna-switching time, tag-reading time, communication failures, or other system-specific sources of missed detections. Therefore, the resulting value should be regarded as a conservative estimate of collision-related data loss and is unlikely to overestimate the practical limitations of LF RFID systems.

Across the entire recording period, 48.7% of all valid mouse-time records involved two or more individuals occupying the same antenna field. The corresponding proportions were 56.4% during the light phase and 40.9% during the dark phase.

The antenna occupancy distribution is summarized in Table S7. Records involving exactly two animals on the same antenna accounted for 33.5% of all valid mouse-time records. Under the single-tag read constraint, one of the two records would be lost, corresponding to a loss of 16.8% of the total dataset. Records involving exactly three animals accounted for 12.5% of all valid records; because two of the three records would be lost, these events contributed an additional loss of 8.3% of the total dataset. Occupancies involving four or more animals were evaluated using the same rule.

Overall, applying this calculation across all occupancy levels yielded an estimated total data-loss rate of 27.1%. The estimated loss rates were 31.9% during the light phase and 22.3% during the dark phase.

These results show that, even under the conservative assumption that one tag is always successfully detected during each multi-tag occupancy, tag collisions alone would result in substantial information loss and preclude direct reconstruction of social co-presence events.

Table S7

Animals per antenna	%All	%Loss All	%Light	%Loss Light	%Dark	%Loss Dark
1	51.30%	0.00%	43.55%	0.00%	59.06%	0.00%
2	33.49%	16.75%	36.38%	18.19%	30.60%	15.30%
3	12.51%	8.34%	15.99%	10.66%	9.03%	6.02%
4	2.47%	1.85%	3.70%	2.77%	1.25%	0.93%
>5	0.22%	0.18%	0.38%	0.30%	0.07%	0.05%
>2	48.70%	27.12%	56.45%	31.93%	40.94%	22.31%

Supplementary Information 8. Rationale for selecting the chattering-correction threshold

S8.1 Distribution of brief return events

To evaluate the appropriateness of the chattering-correction threshold ($\Delta t = 1$ s) used for mice, we analyzed the duration of brief return events before chattering correction.

Using the raw RFID detection logs, antenna positions were tracked over time for each individual. A brief return event was defined as an event in which a tag moved from antenna A to another antenna and subsequently returned to antenna A. For each event, the return duration was defined as the elapsed time between the last detection on antenna A before the switch and the subsequent detection on antenna A after the return.

A total of 648,369 brief return events were identified (Figure S8). The median return duration was 0.526 s, and the 75th, 90th, 95th, and 96th percentiles were 0.616 s, 0.702 s, 0.814 s, and 0.983 s, respectively. Overall, 96.1% of all events returned to the original antenna within 1 s.

These findings indicate that the vast majority of short-duration return events occurred within 1 s and are therefore likely to represent detection fluctuations near antenna boundaries (chattering) rather than genuine locomotor behavior.

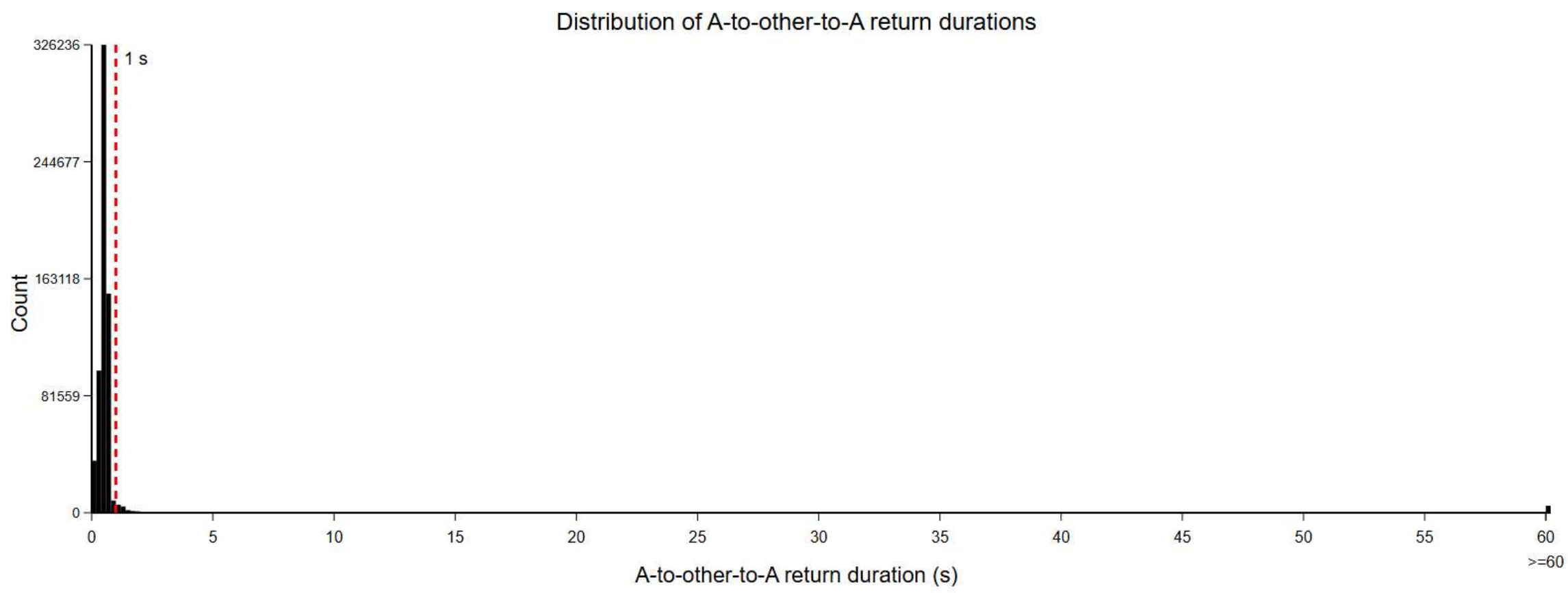

Figure S8. Histogram of return duration

S8.2 Effect of the chattering-correction threshold on follow analysis

To evaluate how the choice of the chattering-correction threshold affected behavioral analyses, the entire follow analysis was independently repeated using $\Delta t = 0.5$, 1.0, and 2.0 s.

For each threshold, follow counts were calculated for all 552 directed mouse pairs. Pair rankings based on follow counts were compared between threshold conditions using Spearman's rank correlation coefficient, together with the Top 25% overlap, defined as the proportion of shared pairs among the highest-ranked 25% (138 pairs) (Table S8).

Between Δt = 0.5 s and Δt = 1.0 s, pair rankings showed only moderate agreement (Spearman's ρ = 0.607), and the Top 25% overlap was 47.1%, indicating that more than half of the highest-ranked follow pairs differed between the two conditions. In contrast, between Δt = 1.0 s and Δt = 2.0 s, pair rankings remained highly consistent (Spearman's ρ = 0.919), with a Top 25% overlap of 83.3%, indicating that the overall follow-network structure was largely preserved.

Together, these results indicate that Δt = 1 s effectively suppresses chattering-derived follow events while preserving the major structure of the follow network.

Table S8

Comparison	Spearman ρ	Top 25% overlap
0.5 vs1	0.607	47.10%
1 vs2	0.919	83.30%
0.5 vs2	0.398	42.00%
